# Response to Letter From Dr. Thorakkal Shamim

**DOI:** 10.5041/RMMJ.10500

**Published:** 2023-04-30

**Authors:** Peter J. Hotez

**Affiliations:** 1 Texas Children’s Hospital Center for Vaccine Development, Department of Pediatrics and Molecular Virology and Microbiology, National School of Tropical Medicine, Baylor College of Medicine, Houston, TX, USA; 2 James A. Baker III Institute of Public Policy, Rice University, Houston, TX, USA; 3 Department of Biology, Baylor University, Waco, TX, USA; 4 Hagler Institute of Advanced Study and Scowcroft Institute of International Affairs, Texas A&M University, College Station, TX, USA

**To the Editor**

Dr. Thorakkal Shamim has written a very interesting letter and comment. It is important to hear details about vaccine hesitancy in different countries or regions. I am especially watchful of our American style of antivaccine activism gaining a foothold abroad. Hence, we need more information about this in the searchable biomedical literature.

I also appreciate Dr. Shamim’s comments about providing accurate and timely vaccine information in our schools. Science education remains a top priority for all free and democratic societies.

